# Supplementary material for: In Vivo Evaluation of the Effects of B-Doped Strontium Apatite Nanoparticles Produced by Hydrothermal Method on Bone Repair
Source: J Funct Biomater. 2022 Jul 31;13(3):110. doi: 10.3390/jfb13030110 (PMC9397061; doi:10.3390/jfb13030110)
Supplement: Supplementary file 1 [file jfb-13-00110-s001.zip › jfb-1830352-supplementary.pdf]

Table S1. 2θ values of diffraction planes.

Sample/ Diffraction planes	200	002	102	210	211	300	310	113	222	312	213	321	402
SrAp	20.99^{o}	24.46^{o}	26.66^{o}	27.90^{o}	30.58^{o}	31.70^{o}	38.35^{o}	41.55^{o}	44.65^{o}	46.04^{o}	46.96^{o}	48.51^{o}	49.95^{o}
SrAp-1B	21.04^{o}	24.51^{o}	26.71^{o}	27.98^{o}	30.63^{o}	31.78^{o}	38.40^{o}	41.55^{o}	44.70^{o}	46.04^{o}	47.01^{o}	48.56^{o}	50.11^{o}
SrAp-5B	21.10^{o}	24.54^{o}	26.74^{o}	28.00^{o}	30.63^{o}	31.84^{o}	38.45^{o}	41.60^{o}	44.73^{o}	46.10^{o}	47.01^{o}	48.62^{o}	50.14^{o}
SrAp-10B	21.15^{o}	24.59^{o}	26.79^{o}	28.04^{o}	30.71^{o}	31.91^{o}	38.51^{o}	41.65^{o}	44.83^{o}	46.17^{o}	47.15^{o}	48.72^{o}	50.22^{o}
